# Knowledge of Infection Prevention and Attitudes Towards HIV/AIDS Among Chinese Dental Bachelor Interns: An Appeal for Educational Intervention

**DOI:** 10.3290/j.ohpd.a44686

**Published:** 2020-07-04

**Authors:** Chang-Xiong Jin, Yu-Chen Meng, Wen-Zhi Du, Dan-Dan Pei, Ang Li

**Affiliations:** a Dentist, Key Laboratory of Shaanxi Province for Craniofacial Precision Medicine Research, College of Stomatology, Xi’an Jiaotong University, Xi’an, China. Performed most of the experiments and wrote the manuscript.; b Master of Dentistry Student, Key Laboratory of Shaanxi Province for Craniofacial Precision Medicine Research, College of Stomatology, Xi’an Jiaotong University, Xi’an, China. Participated in some of the experiments and wrote parts of the manuscript.; c Dentist, Department of Prosthodontics, College of Stomatology, Xi’an Jiaotong University, Xi’an, China. Participated in some of the experiments and wrote parts of the manuscript.; d Associate Professor and Master Tutor, Key Laboratory of Shaanxi Province for Craniofacial Precision Medicine Research, College of Stomatology, Xi’an Jiaotong University, Xi’an, China; Department of Prosthodontics, College of Stomatology, Xi’an Jiaotong University, Xi’an, China. Designed and directed the experiments, and revised the whole manuscript thoroughly.; e Professor and PhD Tutor, Key Laboratory of Shaanxi Province for Craniofacial Precision Medicine Research, College of Stomatology, Xi’an Jiaotong University, Xi’an, China; Department of Periodontology, College of Stomatology, Xi’an Jiaotong University, Xi’an, China. Designed and directed the experiments, and revised the whole manuscript thoroughly.

**Keywords:** attitude, dental students, HIV/AIDS, knowledge

## Abstract

**Purpose:**

The present study aimed to assess the current situation of Chinese dental bachelor interns on HIV/AIDS-related knowledge and their attitudes towards HIV/AIDS patients.

**Materials and Methods:**

A cross-sectional, paper-based survey involving 147 dental students from three Chinese dental schools was conducted. Students were recruited to complete the questionnaire regarding their knowledge, awareness and attitudes concerning HIV/AIDS anonymously and voluntarily.

**Results:**

A total of 144 students responded to the study, generating a response rate of 98.0%. Although 97.0% of the dental students believed dentists were at high risk of HIV infection, 97.2% of students expressed no prejudice towards HIV/AIDS patients and stated their willingness to provide oral healthcare service for such patients. No statistically significant differences in the responses on attitudes and some basic knowledge were found between students who had received the relevant education about infection control and the students who had not. However, regarding most questions about oral manifestations in adult and paediatric patients living with AIDS, the students who received relevant education showed more knowledge than the students who did not (p <0.05). The cognitive level about respecting HIV/AIDS patients’ autonomy and privacy was generally low in all the students.

**Conclusions:**

Most of the dental students in this survey held positive attitudes towards HIV/AIDS patients and good grasp of some basic knowledge about HIV/AIDS. On the other hand, the questionnaire results reflected gaps in education, such as respecting HIV/AIDS patients’ privacy.

Acquired immunodeficiency syndrome (AIDS), which is caused by the human immunodeficiency virus (HIV) attacking vital cells in the human immune system, has become one of the most serious healthcare problems with grievous impact on the infected people and their families.^1^ HIV/AIDS spreads rapidly all around the world, especially in developing countries.^5,30^ In China, the first case was identified in 1985. Over the subsequent decades, the number of new HIV/AIDS infections continued to rise at a high speed.^1,13^ In 2009, the Chinese Ministry of Health proclaimed that an estimated 326,000 people were living with HIV in China and, of these, 107,000 had AIDS.^16^ At the end of 2011, the number of people living with HIV more than doubled, reaching 780,000.^31^ Apparently, a great number of HIV/AIDS infections exist, and new infection is happening every moment.

HIV/AIDS, as one of the most dreaded clinical infectious diseases, can spread person to person by infected blood or body fluids. Among medical care workers, dentists are at high risk for occupational exposure and are more vulnerable to HIV/AIDS infection because HIV can be transmitted by blood-contaminated splash/spatter produced through inhalation of aerosol ejected from handpieces.^8,17^ Furthermore, compared with professional dentists, the risks of accidental exposure among dental interns are higher due to their lack of related knowledge about HIV/AIDS and insufficient standard infection control practices.^18^ A study from the United States announced that 32.8% of dental students had experienced occupational exposures to blood or other potentially infectious instruments, and 39% of these experienced two or more exposures per person.^23^

People living with HIV/AIDS (PLWHA) often suffer from painful oral problems such as candidiasis, hairy leukoplakia and Kaposi’s sarcoma^12,21,22^; those oral lesions exist in as many as 50–70% of all PLWHA, as reported.^12^ It is widely accepted that dentists and dental interns have the ethical responsibility to provide quality dental treatment to PLWHA. However, an unwillingness to treat AIDS patients was expressed by dentists as much as 33.3% of the time in Arab nations^2^ and 39.3% in India,^4^ respectively. One study indicated that only 63% of the 467 surveyed dentists were willing to treat PLWHA in China.^12^ Not only the dentists but also the students may be afraid to offer healthcare to PLWHA. However, except for one study that surveyed the students of Henan Province^13^ and was limited to that region (so the results may not be generalised), scarce data targeting dental students is circulating about the awareness of HIV/AIDS and attitudes towards HIV/AIDS patients in China.

Undergraduate education is the right time for medical students to obtain fundamental knowledge and basic skills for their prospective careers. The undergraduate education for a bachelor’s degree in China takes 5 years. The beginning four years are spent learning theoretical knowledge and basic dental skills practiced in the lab without contact with real patients. Dental students begin to receive clinical training in their fifth year of education as student interns in the clinic. The knowledge and practice habits learned at this stage will undoubtedly affect the attitudes of these dental students towards patients and the quality of oral care they provide in their future work.^19^ Therefore, it is necessary to understand the current situation of dental students in this aspect. The present study aimed to assess the Chinese dental students’ awareness of HIV/AIDS, the knowledge about infection control and attitudes towards HIV/AIDS patients from three universities through a questionnaire survey.

## Materials and Methods

Approval of the present study was obtained from the Ethics Committee of Stomatology Hospital of Xi’an Jiaotong University (No. xjkqll[2018]010). The study was performed in full accordance with the World Medical Association Declaration of Helsinki.

### Participants

A cross-sectional survey was conducted among dental students from three dental schools of Peking University located in northern China (school A), Xi’an Jiaotong University located in middle China (school B) and Wuhan University located in southern China (school C). All the students in Year 5 of the three schools were recruited, and the total number of recruited participants was 147. The questionnaires were distributed among the fifth-year students just at their first days as student interns in clinic. As reported by the students, a lecture lasting 2 h, talking about occupational infection control for dentists, was offered to students in school C beforehand, but no relevant courses were provided to students in the other two universities.

### Data Collection

A self-administered, structured questionnaire, adapted from previous studies,^22^ was used in the present study. The questionnaire consisted of five parts and included a total of 37 questions. Part I focused on the sociodemographic characteristics of the participants. Part II contained eight closed-ended questions about basic knowledge of HIV/AIDS. Part III contained 14 questions and Part IV consisted of four questions about the oral lesions of HIV/AIDS in adults and children individually, with answer options of ‘yes’ or ‘no’. Total scores of Part II, Part III and Part IV were calculated by summing the points awarded for each item. Each correct response scored 1, and incorrect responses scored 0. Part V comprised 11 questions addressing attitudes towards HIV/AIDS or PLWHA. The answer options for these items were agree, neutral and disagree.

Before the questionnaires were distributed, a pilot test was conducted on a random sample of students, and the feedback showed that the questionnaire had good reliability and validity. The participants were invited to answer the questionnaire anonymously and voluntarily in a separate classroom without help and discussion in a limited time of 30 min. The completed surveys were returned to the researchers in sealed envelopes immediately after completion.

### Statistical Analysis

Statistical analysis was conducted using SPSS 17.0 (SPSS, Chicago, IL, USA). The mean knowledge and attitude scores were calculated and compared using one-way analysis of variance (ANOVA). The Fisher’s exact test was used for comparison between groups. P <0.05 was considered statistically significant.

## Results

### Participant Demographics

A total of 147 students were enrolled in the present study. Two students expressed their unwillingness to answer the questionnaires before the study, with no reason. One student dropped out of the survey with an unfinished questionnaire. Finally, 144 participants, of whom 43 were males and 101 were females, completed the study, producing a response rate of 97.9%. As reported by themselves, students from school C (50 students) received related education about HIV/AIDS, but students from school A (36 students) and school B (58 students) did not.

### Basic Knowledge About HIV/AIDS

**Fig 1:**
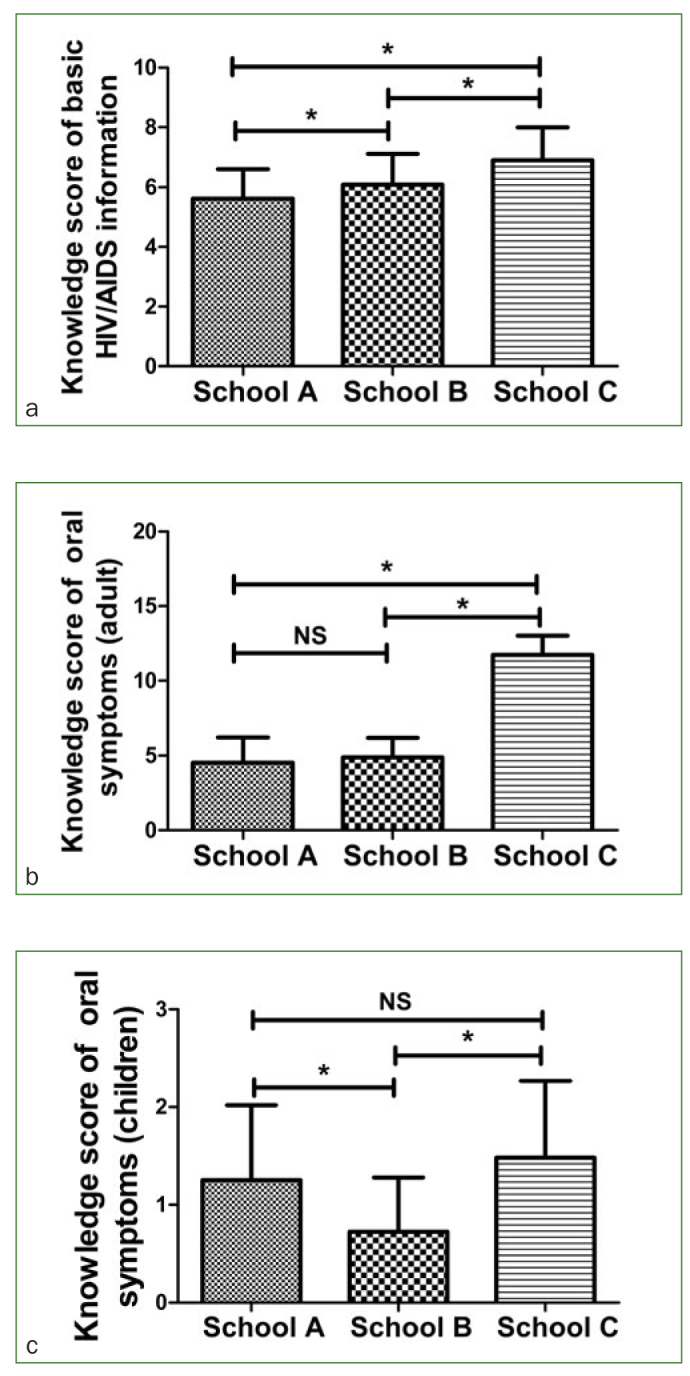
Comparison of the knowledge level of students from three schools. (a) Comparison of students’ knowledge level of basic HIV/AIDS information. (b) Comparison of students’ knowledge level of oral symptoms in adults with HIV/AIDS. (c) Comparison of students’ knowledge level of oral symptoms in children with HIV/AIDS. Data of total knowledge score is presented as means and standard deviations. * p <0.05, NS p >0.05

The survey’s results about students’ basic knowledge about HIV/AIDS appear in Table 1. Of all participants, 100% of the students knew the pathogens of AIDS and 97.2% students knew the mode of transmission. However, only 59.7% of the students knew the length of the HIV incubation period, 86.1% knew the concept of the HIV window period and 66.0% knew the length of the HIV window period. With respect to these three questions, students from school C showed more knowledge than the students from the other two schools (p <0.05). In terms of knowledge on prevention, about 84.7% students declared that ultrasonic dental cleaning was prohibited for HIV-positive patients. A relatively low proportion of students (33.3%) knew the right way to sterilise infective instruments. The average total knowledge scores of participants from the three schools on basic knowledge of HIV/AIDS were compared as shown in Figure 1a. Statistically significant differences were found between any two groups. Students from school C gained the highest mean score of 6.9 among the three groups.

**Table 1:** Correct responses of students to knowledge statements about HIV/AIDS

Statements	Uneducated (%)			Educated (%)	Total (%) (n = 144)	P value
	School A (n = 36)	School B (n = 58)	Total (n = 94)	School C (n = 50)		
Pathogen of AIDS	100.0	100.0	100.0	100.0	100.0	
Modes of transmission	91.7	98.3	95.7	100.0	97.2	>0.05
The length of HIV incubation period	47.2	43.1	44.7	88.0	59.7	<0.05
The concept of HIV window period	61.1	91.4	79.8	98.0	86.1	<0.05
The length of HIV window period	61.1	58.6	59.6	78.0	66.0	<0.05
Ultrasonic dental cleaning for patients with HIV/AIDS	77.8	91.4	86.2	82.0	84.7	>0.05
Cleaning and disinfection of the contaminated instruments	22.2	31.0	27.7	44.0	33.3	>0.05
Wearing gloves when doing the oral examination	100.0	94.8	96.8	100.0	97.9	>0.05

P value indicates comparison of the correct response percentage between students who received relevant education about infection control (school C) and students who did not (school A and school B), using the Fisher’s exact test.

### Knowledge of Oral Lesions in Adults with HIV/AIDS

The results about students’ knowledge of oral lesions in adult PLWHA are shown in Table 2. Of 14 questions on this topic, the correct-answer rate of 12 questions was lower than 68.0%. Only two questions on oral candidiasis and periodontitis gained 88.1% and 86.1% correct answering rates, respectively. On each of the 14 questions regarding oral lesions in adult PLWHA, students from school C showed more knowledge than did students from the other two schools (p <0.05), and students from school C gained the highest total score of 11.7 among the three groups (Fig 1b). There was no statistically significant difference between school A and school B (p >0.05).

**Table 2:** Correct responses of students to knowledge statements about oral symptoms in adults with HIV/AIDS

Oral symptom (adult)	Uneducated (%)			Educated (%)	Total (%) (n = 144)	P value
	School A (n = 36)	School B (n = 58)	Total (n = 94)	School C (n = 50)		
Oral candidiasis	83.3	82.8	83.0	98.0	88.2	<0.05
Necrotic stomatitis	38.9	8.6	20.2	78.0	40.3	<0.05
Hairy leukoplakia	30.6	67.2	53.2	96.0	68.1	<0.05
Linear gingival erythema	19.4	69.0	50.0	96.0	66.0	<0.05
Necrotising periodontitis	25.0	13.8	18.1	80.0	39.6	<0.05
Periodontitis associated with HIV	77.8	81.0	79.8	98.0	86.1	<0.05
Acute necrotic ulcerative gingivitis	27.8	19.0	22.3	86.0	44.4	<0.05
Kaposi’s sarcoma	30.6	63.8	51.1	94.0	66.0	<0.05
Recurrent aphthous ulcer	11.1	12.1	11.7	68.0	31.3	<0.05
Salivary gland diseases	8.3	5.2	6.4	68.0	27.8	<0.05
Histoplasmosis	11.1	5.2	7.5	86.0	34.7	<0.05
Non-Hodgkin lymphoma	19.4	19.0	19.2	86.0	42.4	<0.05
Oral herpes	52.8	13.8	28.7	74.0	44.4	<0.05
Papillary epithelioma, focal epithelial hyperplasia	13.9	19.0	17.0	66.0	34.0	<0.05

P value indicates the comparison of the correct response percentage between students who received relevant education about infection control (school C) and students who did not (school A and school B), using the Fisher’s exact test.

### Knowledge of Oral Lesions in Children with HIV/AIDS

Table 3 shows students’ correct-answer rates to each question about oral lesions in children PLWHA. Oral candidiasis was known by more than half of the students from different schools (69.4% in school A, 56.7% in school B and 80.0% in school C, respectively). However, regarding angular cheilitis, oral candidiasis and herpes simplex, only about 15.0% or fewer students gave correct responses in total. Except for angular cheilitis, students from school C had a much higher correct-answer percentage than students from the other two schools in the other three items (p <0.05). The comparison results of total knowledge scores in this part are shown in Figure 1c. School A and school C showed a comparable knowledge level in this part (p >0.05). Students from both school A and school C gained higher scores than students from school B (p <0.05).

**Table 3:** Correct responses of students to knowledge statements about oral symptoms in children with HIV/AIDS

Oral symptom (children)	Uneducated (%)			Educated (%)	Total (%) (n = 144)	P value
	School A (n = 36)	School B (n = 58)	Total (n = 94)	School C (n = 50)		
Angular cheilitis	22.2	5.2	11.7	14.0	12.5	>0.05
Oral candidiasis	69.4	56.7	61.7	80.0	68.1	<0.05
Parotitis	16.7	3.5	8.5	30.0	16.0	<0.05
Herpes simplex	16.7	6.9	10.6	24.0	15.3	<0.05

P value indicates the comparison of the correct response percentage between students who received relevant education about infection control (school C) and students who did not (school A and school B), using the Fisher’s exact test.

### Attitude Towards HIV/AIDS or PLWHA

The attitude distributions of students towards HIV/AIDS or PLWHA are presented in Table 4. The majority (97.2%) expressed optimistic attitudes towards PLWHA. Meanwhile, 92.4% of students responded with a willingness to provide treatment for patients with HIV/AIDS. Although 96.5% of students thought that dental workers are at high risk of HIV infection, 100% of students supported learning and spreading knowledge about HIV, and 77.8% of students showed their fearlessness of HIV/AIDS. To reduce the possibility of HIV infection, 72.2% of students insisted that all patients should have a blood test before oral therapy, and 86.8% of students felt that HIV/AIDS patients should disclose their disease to other people. No statistically significant differences in the responses were found between students from school C, who had received relevant education, and students from school A and school B, who had not.

**Table 4:** Attitudes to patients with HIV/AIDS or HIV/AIDS

Attitude statements	Uneducated (%)			Educated (%)	Total (%) (n = 144)	P value
	School A (n = 36)	School B (n = 58)	Total (n = 94)	School C (n = 50)		
**Caring and not discriminating against patients with HIV**						
Agree	94.4	100.0	97.9	96.0	97.2	>0.05
Neutral	2.8	0.0	1.1	4.0	2.1	
Disagree	2.8	0.0	1.1	0.0	0.7	
**Willing to treat patients with HIV/AIDS**						
Agree	94.4	88.0	90.4	96.0	92.4	>0.05
Neutral	2.8	7.0	5.3	2.0	4.2	
Disagree	2.8	5.2	4.3	2.0	3.5	
**Learning and disseminating knowledge about HIV/AIDS**						
Agree	100.0	100.0	100.0	100.0	100.0	
Neutral	0.0	0.0	0.0	0.0	0.0	>0.05
Disagree	0.0	0.0	0.0	0.0	0.0	
**Oral medical personnel are susceptible to HIV/AIDS**						
Agree	86.1	100.0	94.7	100.0	96.5	>0.05
Neutral	2.8	0.0	1.1	0.0	0.7	
Disagree	11.1	0.0	4.3	0.0	2.8	
**Patients with HIV/AIDS should disclose it**						
Agree	88.9	87.9	88.3	84.0	86.8	>0.05
Neutral	5.6	5.2	5.3	6.0	5.6	
Disagree	2.8	6.9	6.4	10.0	6.9	
**A blood test should be taken in all dental patients**						
Agree	94.4	67.2	77.7	68.0	72.2	>0.05
Neutral	5.6	6.9	6.4	4.0	5.6	
Disagree	0.0	25.9	16.0	28.0	22.2	
**Infectious disease education for all patients**						
Agree	91.7	100.0	96.8	96.0	94.4	
Neutral	8.8	0.0	3.2	2.0	2.8	>0.05
Disagree	0.0	0.0	0.0	2.0	2.8	
**Not afraid of HIV/AIDS**						
Agree	55.6	89.7	76.6	80.0	77.8	>0.05
Neutral	2.8	0.0	1.1	2.0	1.4	
Disagree	41.7	10.4	22.4	18.0	20.8	
**HIV/AIDS is far from us**						
Agree	0.0	0.0	0.0	0.0	0.0	>0.05
Neutral	5.6	0.0	2.1	0.0	1.4	
Disagree	94.4	100.0	97.8	100.0	98.6	

P value indicates the comparison of the attitude choice percentage between students who received relevant education about infection control (school C) and students who did not (school A and school B), using the Fisher’s exact test.

## Discussion

Exposure to blood-borne pathogens such as HIV is considered a challenge for healthcare workers, especially in developing countries such as China, where the health settings are not advanced enough.^10,26^ Sources of fear of providing care for PLWHA mostly stem from overestimation of the virus transmission risk.^7^ Compared with other studies,^28^ it was satisfying to find that more than 97.0% of participants had accurate knowledge about transmission modes of HIV/AIDS. Only several students had misconceptions about how HIV is transmitted. They mistakenly believed that HIV could spread by sharing public facilities, shaking hands or acquiring mosquito bites, which had been addressed by previous studies.^17,20^

The window period of HIV, which may reach up to 3 months, is a special stage referring to a time period following an infection, during which antibodies against HIV are not detectable.^33^ The incubation period is the period between the exposure to HIV pathogen and the appearance of symptoms of any disease indicative of AIDS.^25^ Patients in both the HIV window period and incubation period have strong infectivity. Although more than 85.0% of students knew the concept of the window period, only 66.0% knew the length of it, and only 59.7% of students knew the length of the incubation period of HIV. With respect to this information, students from school C responded with more correct answers than did students from the other two schools (p <0.05), indicating the positive effect of infection control education on students.

Oral lesions were considered one of the earliest and reliable clinical indicators of HIV infection.^24^ Patients may not realise that they have been infected with HIV until some oral symptoms occur, which makes them go for further confirming tests. Hence, dental workers should be aware of these oral manifestations to assist early diagnosis of AIDS in those vulnerable populations. Up to now, as many as 40 kinds of oral manifestations related to HIV infection have been identified.^12,21^ Results in Table 1 and Table 2 show that the students from school C recognised more oral lesions related to HIV infection than did students from the other two schools. However, in general, except for oral candidiasis and periodontitis associated with HIV patients, the other 12 symptoms surveyed in the present study were only recognised by less than 68.0% of all the respondents from three schools, which is less than the results reported by Sadeghi and Hakimi among Iranian dental students^27^ and the study conducted by Singh et al among Malaysia dental students.^29^

Discrimination and stigma against people with HIV/AIDS is fuelling the spread of the epidemic and making healthcare workers unwilling to provide treatment for patients.^32^ People’s fear of AIDS usually comes from misconception of HIV/AIDS and the currently incurable characteristics of this disease^11^; 77.8% of students reported that they were not afraid of HIV/AIDS, but 20.8% admitted their fear, which is similar to previous reports.^3,11^ In the present survey, despite 96.5% of students believing that dentists were at high risk of infection, 97.2% of students expressed no prejudice towards PLWHA, and 92.4% expressed their willingness to provide therapy for them, which is higher than the proportions reported by the previous studies.^10,14^

Dentists are legally obligated to respect the autonomy and privacy of their patients. Autonomy gives patients the right to refuse an HIV test, and privacy guarantees patients freedom to decide whether to disclose their HIV status.^15,34^ Moreover, people with HIV infection are not requested by law to disclose their HIV status to any healthcare professionals in China. Students in the present study showed poor understanding of this aspect; 72.2% students declared that all patients should take a blood test before accepting treatment, and around 86.8% students thought that it was necessary for PLWHA to disclose their disease. Undoubtedly, those demands may cause huge psychological pain to HIV/AIDS patients. Hence, respecting patients’ rights should be paid more attention, and all medical care workers should give enough humane care to PLWHA in the process of their diagnosis and treatment of patients.

School education was identified as a key promoting factor to improve students’ cognition of HIV/AIDS.^6,9^ A study from Ethiopia reported that students who had attended an educational class about HIV/AIDS had more integrated knowledge of HIV/AIDS than did those who had not acquired such education.^20^ In the present study, students from school C who received some HIV/AIDS-related education showed more knowledge of the oral manifestations of AIDS, the window period and the incubation period of HIV. These results demonstrated that an education programme is effective in significantly improving knowledge, which was also proven by other studies.^6,12^ However, the cognitive level about respecting patients’ autonomy and privacy was generally low. No statistically significant differences were found between students who received education or did not before. This reflected the gap in the education contents. Therefore, expanding and deepening HIV/AIDS-related education, especially on occupational protection and the particularities of providing medical service to PLWHA, is greatly needed for dental students in China for future progress.

## Conclusion

The present study investigated the current cognition level of dental bachelor interns in China. Although three representative schools from different locations were included in the present survey, the results could not be generalised to all preclinical dental students. Thus, further research is needed with larger sample sizes. In addition, the study was a cross-sectional study; therefore, pre- and posteducational surveys are welcomed in the future with a randomised controlled trial design. Within the limitation of the present study, the results indicate that infection control education might play a vital role in promoting knowledge of HIV/AIDS and, at the same time, reflect the gap in the education contents, such as information about respecting HIV/AIDS patients’ autonomy and privacy. HIV/AIDS infection control-related courses are not a required course in all the dental schools in China. Therefore, the present study raises a call for educational intervention on HIV/AIDS infection control. Moreover, the education content should be designed meticulously to provide comprehensive and useful knowledge for dental students.
